# Nudging Healthier and More Sustainable Eating Habits in University Cafeterias: The FOOD-HACK Project

**DOI:** 10.3390/nu17223562

**Published:** 2025-11-14

**Authors:** Sara Basilico, Ilaria Zambon, Rachele De Giuseppe, Lidia Testa, Andrea Del Bo, Veronika Gamper, Valentina Moroni, Maria Elide Vanutelli, Hurisel Tosun, Htoi Lu Mai Hpau Yam, Maria Vittoria Conti, Hellas Cena

**Affiliations:** 1 Laboratory of Dietetics and Clinical Nutrition, Department of Public Health, Experimental and Forensic Medicine, University of Pavia, 27100 Pavia, Italy; ilaria.zambon01@universitadipavia.it (I.Z.); rachele.degiuseppe@unipv.it (R.D.G.); hurisel.tosun01@universitadipavia.it (H.T.); htoilumai.hpauyam01@universitadipavia.it (H.L.M.H.Y.); mariavittoria.conti@unipv.it (M.V.C.); hellas.cena@unipv.it (H.C.); 2 MARKAS Italia S.r.l., 39100 Bolzano, Italy; 3 Department of Psychology, University of Milan-Bicocca, 20126 Milan, Italy; maria.vanutelli@unimib.it; 4 Operational Unit of Clinical Nutrition, Istituti Clinici Scientifici Maugeri, Istituto di Ricovero e Cura a Carattere Scientifico, 27100 Pavia, Italy; 5 Italian Institute for Planetary Health, 20156 Milan, Italy

**Keywords:** nudging, university cafeteria, nutrition, Mediterranean diet, Planetary diet, health, sustainability

## Abstract

**Background/Objectives**: The global syndemic of obesity, undernutrition, and climate change highlight the complex health and environmental challenges faced by young adults. These challenges may intensify during the transition to university. As a matter of fact, limited budgets, time constraints, and insufficient culinary skills often lead to unbalanced diets and increased risk of obesity. University cafeterias, serving large numbers of students, represent an ideal setting to promote healthier and more sustainable eating behaviors. The FOOD-HACK Project aimed to design and implement a cafeteria-based intervention using nudging strategies to promote healthier and more sustainable lunch choices among university students. **Methods**: This pilot study employed a pre–post design with two independent phases in the Polo Cravino cafeteria at the University of Pavia. Food consumption was assessed over 12 non-consecutive days across four weeks. During the intervention, three nudging strategies were implemented: (1) choice architecture, (2) salient labeling, highlighting healthy and sustainable options, and (3) educational prompts. **Results**: Across both phases, 2400 tray photographs were collected. Post-intervention, the proportion of trays aligned with the Harvard Healthy Eating Plate and EAT-Lancet Planetary Diet models increased, reflecting higher consumption of vegetables and fruit. Legume-based first courses increased; however, legumes did not substantially replace animal proteins as the main protein source, and meat remained predominant in second courses. Reductions in trays containing multiple carbohydrate sources were also observed. **Conclusions**: The nudging intervention improved overall meal quality, demonstrating that subtle environmental modifications can guide students toward healthier dietary choices, particularly by increasing fruit and vegetable intake. However, the persistent preference for animal proteins highlights the challenge of shifting protein consumption toward more sustainable sources. These findings suggest that nudging can be an effective tool to promote healthier and more balanced eating behaviors in university settings, though complementary strategies may be needed to foster substantial changes in protein choices.

## 1. Introduction

The intertwined crises of obesity, undernutrition, and climate change form a global syndemic that exposes the urgent need to address the shared determinants of human and environmental health [1]. Unhealthy dietary habits not only contribute to the rising prevalence of obesity but also exacerbate environmental degradation and widen social inequalities, highlighting the urgent need for integrated, sustainable solutions [1]. University students, as a key demographic group, experience significant lifestyle transitions during their academic years. These changes, often marked by limited budgets, time constraints, and insufficient cooking skills, frequently lead to unbalanced eating patterns [2]. As noted by Arnett et al. [3], young adulthood represents a developmental stage characterized by physiological, social, and cultural transformations, including leaving the family home and being influenced by peer groups, both of which can reinforce unhealthy habits.

University students often exhibit a high consumption of processed and ultra-processed foods and a low adherence to healthy, balanced, and sustainable dietary models such as the Mediterranean Diet (MD) [4].

Maintaining nutritionally adequate eating habits during this life stage is essential, as food choices established in young adulthood tend to persist into later life, affecting long-term health outcomes [5]. However, evidence shows that university students typically consume fewer portions than recommended from key food groups, such as cereals, fruits, vegetables, dairy and legumes, while favoring high-fat and sugary foods [6]. These poor eating patterns are associated with increased risks of obesity, metabolic syndrome, and other non-communicable diseases (NCDs), reinforcing the urgency of early interventions that can effectively promote healthier behaviors [2,7].

Against this backdrop, nudging, a strategy grounded in behavioral economics, has emerged as an effective approach to gently influence food choices without restricting individual freedom [7,8]. By subtly modifying the food environment, nudging leverages cognitive biases and decision-making shortcuts to encourage healthier and more sustainable choices [9]. Several nudging techniques have proven effective across different populations, including university students [10,11,12,13]. Among the most commonly applied in food service settings are choice architecture (enhancing the visibility and accessibility of healthy items) [8,14], salient labeling (strategic placement of descriptive or attention-grabbing labels) [15] and educational prompts (providing brief health-related information to guide choices) [13]. For example, positioning fruits and vegetables at eye level or along high-traffic routes increases their uptake, while creative, appealing dish names foster more positive attitudes towards nutritious options [16].

Given that, many students rely on university cafeterias for one or more daily meals [17], these settings are especially well-placed to shape eating behaviors and to apply nudging strategies within a large and diverse student population. Cafeterias offer a controllable environment where small, systematic adjustments can yield measurable changes in selection patterns towards healthier and more sustainable choices [13]. In Italy, for instance, Cesareo et al. [16] implemented a cafeteria-based nudging program at the University of Milano-Bicocca that used both choice architecture and salient labeling by prominently displaying whole-grain bread and fresh fruit, and by adding visible symbols to identify healthier options (e.g., legumes, vegetables, and plant-based dishes).

Complementing these health-oriented cafeteria nudges, there is a parallel imperative to align dietary interventions with environmental sustainability. Growing concerns about resource depletion, climate change, food insecurity, and diet-related health issues, together with the social and environmental consequences of food production and consumption, have led policymakers to design new strategies aimed at promoting healthier and more sustainable eating habits [18,19]. Dietary models such as the Healthy Eating Plate developed by the Harvard T.H. Chan School of Public Health [20] and the Planetary Diet proposed by the EAT-Lancet Commission [21] exemplify this approach, as they have the potential to benefit both human health and the planet. Evidence shows that foods like legumes, nuts, seeds, fruits, and vegetables not only contribute to better health outcomes but also tend to have a lower environmental footprint than most animal-based products, making plant-based dietary patterns particularly promising for sustainability and well-being [21,22].

Yet, traditional food services often offer few appealing plant-based alternatives to meat and fish dishes. Expanding the availability and visibility of vegetarian options has a substantial effect on shifting consumer choices [23,24]. In some studies, doubling the number of plant-based options resulted in a 38% increase in their selection, thereby reducing greenhouse gas emissions associated with meat production [23].

Other emerging strategies include digital applications that display the environmental footprint of menu items in real time, enabling students to make informed and conscious choices [25,26,27]. Likewise, initiatives such as “Meatless Day” campaigns have gained traction in encouraging students to reflect on the environmental impact of their food choices [23].

Extending these cafeteria-wide and sustainability-oriented measures, micro-level cues can further reinforce healthier, lower-impact choices at the moment of decision-making. Subtle environmental signals (such as brightly colored signage highlighting healthy options or posters showcasing traditional dishes prepared with fresh ingredients) can effectively capture attention and nudge selections towards healthier and more sustainable alternatives. For instance, some studies have used specially designed plates to encourage balanced meal composition. One study examined the impact of a plate with color-coded sections indicating appropriate portions for each food group [28], while another tested a graphical representation of a healthy plate (half filled with vegetables) to promote greater vegetable intake [29]. Moreover, another intervention [30] reformulated canteen menus based on the optimal meal composition outlined in the Harvard Healthy Eating Plate [20]. A dedicated “Healthy and Sustainable” logo likewise serves as a rapid visual heuristic, enabling students to identify dishes that satisfy health and sustainability criteria at a glance [16]. These approaches align with evidence from cognitive psychology indicating that people often struggle with information overload in everyday choices [31,32,33,34]. By offering simplified prompts, including logos, colors, and other salient markers, cafeterias reduce cognitive load and engage intuitive decision processes, thereby facilitating healthier and more sustainable selections [35].

Building on this framework, the Food-Hack Project “One small nudge for a cafeteria, one giant impact for students: Fostering Healthier Eating Habits in University Cafeterias” is a pilot study conducted at the university of Pavia (Italy), which aimed to explore how subtle nudging strategies can promote healthier and more sustainable food choices among university students. The project aimed to reshape the cafeteria environment to make balanced and plant-forward meal selections easier and more appealing, drawing inspiration from the Harvard Healthy Eating Plate [20] and the Planetary Health Diet proposed by the EAT-Lancet Commission [21]. Specifically, the intervention sought to increase the selection of plant-based Mediterranean diet components (e.g., vegetables, legumes, whole grains, and fruit) and to encourage the composition of nutritionally balanced meals aligned with both human and planetary health principles. Using a pre–post evaluation over 12 non-consecutive days, the study assessed changes in food item selections, with the goal of a scalable model for university cafeterias.

## 2. Materials and Methods

### 2.1. Study Objective

The primary aim of this study was to design and implement a cafeteria-based intervention using nudging strategies to promote healthier and more sustainable lunch choices among university students. The intervention specifically targeted an increased selection of MD plant-based components (e.g., vegetables, legumes, whole grains, and fresh fruit) and encouraged the composition of balanced meals consistent with the Harvard Healthy Eating Plate and the Planetary Health Diet frameworks [20,21].

### 2.2. Partnership

The current pilot study was implemented at one of the cafeterias (Polo Cravino) of the University of Pavia. The cafeteria was selected based on practical considerations, including its accessibility and logistical feasibility for implementing the intervention. The study was designed by the Laboratory of Dietetic and Clinical Nutrition (LDNC) in collaboration with the Office for Sustainable Actions (OSA) of the University of Pavia and with Edisu, Ente per il diritto allo Studio Universitario (the Regional Agency for the Right to Higher Education) and Markas Italia s.r.l., leading companies in collective catering services.

#### 2.2.1. Laboratory of Dietetic and Clinical Nutrition

The LDNC is a multidisciplinary research group with national and international expertise in the fields of dietetics and clinical nutrition, lifestyle medicine, and personalized nutrition. The group’s research has focused on malnutrition, both undernutrition and overnutrition related to non-communicable diseases, with particular attention to vulnerable populations. LDNC is also engaged in the study and promotion of biodiversity and sustainability as key factors in supporting and preserving individual well-being, in line with the goals of the 2030 Agenda (Sustainable Development Goals, SDGs) [36]. Within the FOOD HACK project, LDNC researchers, as members of OSA in the working group “Health and Well-being”, acted as Principal Investigators (P.I.). In particular, the researchers identified the nudging strategies, developed the content of the posters, and conducted the operational phase of data collection.

#### 2.2.2. Office for Sustainable Actions

OSA gathers, develops, and implements best practices, fosters new initiatives, and prepares strategic documents to enhance awareness and engagement on sustainability topics within the university community and the wider public. Aligned with the 17 SDGs, OSA focuses on six main areas: infrastructure and green spaces, energy, waste and circular economy, mobility, social sustainability, health and well-being, while integrating sustainability into teaching, research, and third mission activities. Within the FOOD HACK Project, OSA acted as P.I., defining the sustainability components to be integrated into the nudging strategies. OSA also served as a liaison between LDNC and the catering company Markas Italia S.r.l.

#### 2.2.3. Edisu and Markas Italia s.r.l.

At the University of Pavia, catering services are provided through a dual system that ensures broad accessibility and flexibility. On one hand, EdiSU directly manages several on-campus dining facilities, primarily offering lunch services to meet students’ daily needs. On the other hand, the University also relies on external catering companies, such as Markas Italia s.r.l. (Manager of the Polo Cascina Cravino university cafeteria), through formal agreements and service contracts, to further expand the dining options available. Within this study, the company provided its marketing office to produce the graphic materials for the nudging intervention.

### 2.3. Study Population and Setting

The selected cafeteria operates daily from 12:00 p.m. to 2:00 p.m., offering 200 seats and serving an average of 350–400 meals per day. The primary users are university students, although professors and administrative staff may also access the facility. For this study, only individuals who consumed their meals on-site were included in the sample, while those who purchased food for take-away were excluded.

### 2.4. Experimental Design

**Figure 1:**
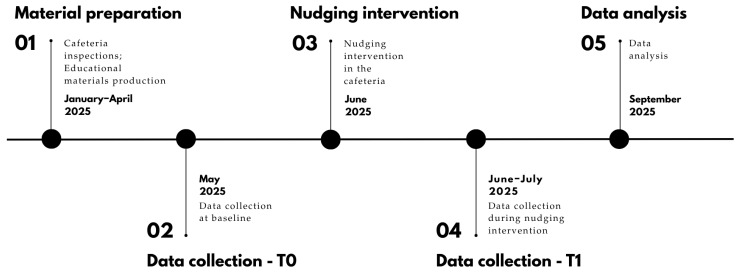
Timeline of the FOOD-HACK project.

The present pilot study employed a pre–post design with two distinct phases. The independent variable was the implementation of nudging strategies during the post phase, selected to assess intervention effects while preserving routine canteen operations. The primary outcome was the selection of predefined target foods, one for each course of a typical Italian meal (including first course, second course, side dishes, bread, and fruit/dessert). Conducted in a real-world setting, this design enabled evaluation of effectiveness with high practical relevance and transferability to similar contexts. The study timeline is reported in Figure 1.

#### 2.4.1. Data Collection (T0 and T1)

The observational phase, both at T0 and at T1, was carried out on Mondays, Wednesdays, and Fridays over four weeks (12 service days) from 5 May to 30 May 2025 and from 23 June to 18 July, respectively. Non-consecutive days were deliberately chosen to ensure variability in meal options and consumption patterns, thereby providing a more representative overview of students’ dietary behaviors across the week. During each observation day, two trained researchers stationed at the point of sale (adjacent to the cashier) systematically photographed diners’ trays immediately after meal selection and before consumption, yielding 200 usable tray photographs at T0 and at T1. Images excluded any personal identifiers; no individual-level demographic data were recorded.

All photographs were imported into a dedicated database and coded according to a pre-specified data dictionary. For each tray, every visible component was recorded and assigned to one of six categories reflecting a typical Italian meal structure: first course; second course; side dish; bread; fruit/dessert; and beverage [37].

After the first data collection (T0), the nudging intervention was implemented in the canteen space. The second data collection (T1) started three weeks after the implementation to allow students to adapt to the new environment.

The project targeted the summer four-week rotating menu offered by the cafeteria. Table S1 shows the full menu available during the data collection days (Mondays, Wednesdays, and Fridays). Typically, two to three options were available for first courses, second courses, and side dishes, and four options for the end-of-meal course. In addition, a few fixed alternatives were always offered (e.g., plain pasta or rice, grilled meat, fish, or vegetables).

**Figure 2:**
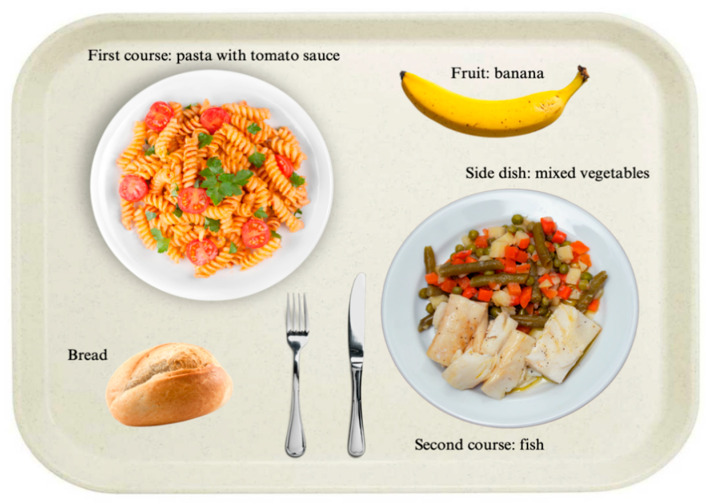
Example of try with an Italian meal composition.

To better clarify the composition of a typical Italian meal, Figure 2 shows an example of a tray.

According to the menu (Table S1) and the cafeteria policy, a tray can be composed as follows:

- Option 1: One dish of first course, one dish of second course, one or more side dishes, bread and one or two servings of either fruit or dessert.
- Option 2: Two first courses, one or more side dishes, bread and one or two servings of either fruit or dessert.
- Option 3: Two second courses, one or more side dishes, bread and one or two servings of either fruit or dessert.
- Option 4: Pizza, one or more side dishes, and one or two servings of either fruit or dessert.

Each customer could compose their tray among the options (option 1–option 4) listed above. Since trays were registered at the cashier upon payment, it was inferred that each tray corresponded to a single customer’s meal.

#### 2.4.2. Data Codification

Category-specific coding criteria captured dish composition (e.g., dominant protein/source and cereal type) and preparation features where discernible. For first courses, items were classified by dominant ingredient into meat-based, fish-based, vegetable-based, or legume-based preparations; pasta/grain type (whole-grain vs. refined) was additionally noted when visually ascertainable.

Second courses were classified by primary protein source (meat, fish/seafood, eggs, cheese, or plant-based/legume-derived). Side dishes were coded for vegetable presence (yes/no) while bread was coded by grain type (whole-grain vs. refined; whole-grain defined as predominantly whole-meal flour). Fruit/dessert was recorded for presence (yes/no), and beverages were categorized as water or sugar-sweetened (containing caloric sweeteners).

Two independent raters performed coding following a brief calibration on a sample set; a random 10% of records were double coded to assess inter-rater reliability (Cohen’s κ), with discrepancies resolved by consensus and, when necessary, adjudication by a third reviewer. Quality control included periodic checks for internal consistency and completeness before database lock.

Based on the aforementioned category-specific criteria, tray-level dietary patterns were identified as follows: omnivore (e.g., any selection containing meat, fish/seafood, or other animal-source foods), vegetarian (e.g., no meat or fish/seafood selected, but eggs and/or dairy permitted), and vegan (e.g., no animal-source items selected, i.e., excludes meat, fish/seafood, eggs, and dairy). This classification was derived directly from the observed composition and preparation features recorded for each tray.

As a gold standard for the ideal tray composition, we referred to the optimum meal composition of the Harvard Healthy Eating Plate [20] and to the principles of the and the EAT-Lancet Planetary Diet [21]. Together, these models emphasize the importance of a balanced and sustainable meal consisting of approximately 50% fresh fruits and vegetables, 25% cereals (preferably whole grains), and 25% healthy protein sources, with particular attention to increasing the proportion of plant-based proteins. Accordingly, trays were considered compliant with these models [20,21] if they included one source of carbohydrates (pasta, bread, or potatoes), one source of protein (meat, fish, legumes, eggs, or cheese), and at least one serving of vegetables and fresh fruit. Additionally, based on these guidelines, six negative dietary quality indicators were defined:

- Excessive animal protein: trays containing both meat and fish, or double portions of either.
- Absence of protein: trays lacking any protein source (meat, fish, legumes, eggs, cheese).
- Absence of vegetables: trays without a vegetable side dish.
- Excessive carbohydrate sources: trays including pasta or rice together with bread and potatoes.
- Absence of fruit: trays without fruit at the end of the meal.
- Absence of water: trays without water (tap or bottled), where only other beverages were selected.

Pizza orders were placed directly at the cashier, after which customers collected their pizzas at the oven station. As a result, at the time of photographing the trays, the pizzas were not yet present, making it impossible to determine the type of toppings added (e.g., plain margherita, or with additional ingredients such as cured meats or fries). For this reason, trays containing pizza were excluded from analyses concerning dietary patterns and adherence to both the Harvard Healthy Eating Plate [20] and the EAT-Lancet Planetary Diet models [21].

#### 2.4.3. Nudging Strategies Implementation

The nudging strategies aimed to promote the selection of the plates to compose a healthy and sustainable meal, enhancing the consumption of vegetables, fresh fruit and plant-based proteins. To incentivize the selection of the healthiest choice, three integrated nudging approaches were implemented as follows:

(1) Increasing ease/convenience (choice architecture): the central counter, previously displaying desserts (fresh fruit and sweet items together), was reorganized to present only fresh fruit. Sweet desserts remained available but were relocated to a separate, less prominent area.
(2) Providing disclosures (salient labeling): plant-based dishes (Figure S1) and legumes (Figure S2) were flagged with distinctive tags to enhance salience and encourage uptake. Tags for plant-based items read “Best Choice: plant-based and sustainable”; legume dishes were labeled “Reminder: legumes are the protein source of your dish, not a side dish”.
(3) Priming (educational prompts): posters, flyers, and “talking” placemats highlighting the benefits of the Mediterranean Diet and sustainable eating were positioned along queue lines and adjacent to targeted items. Materials were displayed in Italian; each poster also included a QR code (“Scan me for the English version”) linking to an English translation to ensure accessibility for non-Italian-speaking students.

Concerning the educational materials realized, along the diner’s path, the team positioned a coordinated set of educational prompts to guide choices without interrupting service flow. At the canteen entrance, a large sustainability poster introduced the theme by depicting three “footprints” (including greenhouse-gas emissions, water use, and land use) and showing, proportionally within each, the environmental impact of major food groups (meat, fish, legumes, eggs, dairy, vegetables, grains), (Figure S3) [38,39].

As students approached the service line, two “healthy plate” posters, inspired by the optimum meal composition of the Harvard Healthy Eating [20], presented the components of a balanced, nutrient-dense meal and were displayed next to the weekly menu to support planning before selection (Figure S4). Closer to specific choice points, concise flyers reinforced targeted behaviors. Next to the bread baskets, messages encouraged choosing whole grain over white bread for combined health and sustainability benefits (Figure S5) [40,41].

At the fruit counter, a flyer promoted fresh, seasonal fruit as an appropriate way to conclude the meal (Figure S6) [42]. Near both the vegetable counter and the main-course station, paired flyers emphasized the nutritional value of legumes and framed them as a primary protein source rather than a side dish, normalizing plant-based choices (Figure S7) [43]. At the condiments station, guidance on using herbs and spices highlighted flavor enhancement while reducing salt use (Figure S8) [44,45]. By the two water-refill points and next to the beverages counter, prompts advocated water as the default mealtime beverage and encouraged reusable bottles to minimize plastic waste (Figure S9) [46]. Finally, a tray placemat echoed the healthy-plate concept with practical tips for composing a balanced and sustainable meal, ensuring message reinforcement during consumption (Figure S10) [20].

Overall, the intervention embedded sustainability and health into everyday cafeteria decisions, advancing campus-level implementation of multiple goals of the 2030 Agenda, in particular SDG 3 (Good Health and Well-being), SDG 2 (Zero Hunger, targets on safe and nutritious food), SDG 12 (Responsible Consumption and Production), SDG 13 (Climate Action), and SDG 4 (Quality Education) [36].

**Figure 3:**
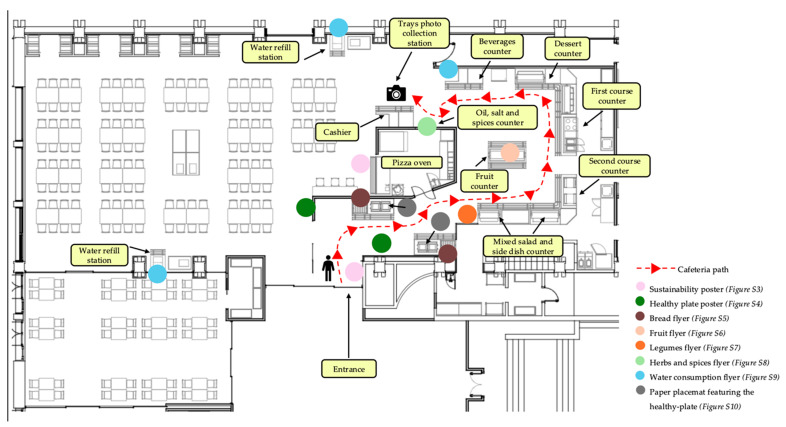
Map of the cafeteria indicating the placement of nudging materials. Sustainability poster (Figure S3); healthy plate poster (Figure S4); Bread flyer (Figure S5); Fruit flyer (Figure S6); Legumes flyer (Figure S7); Herbs and spices flyer (Figure S8); Water consumption flyer (Figure S9); Paper placemat featuring the healthy plate (Figure S10).

Figure 3 provides a schematic representation of the cafeteria layout, indicating the strategic placement of nudging materials and the typical path taken by students from food selection to payment.

### 2.5. Ethical Considerations

During the study period, notices were posted at the cafeteria entrance (adjacent to the menu) and at each cash register. They informed customers that two researchers would photograph the food on trays for research purposes, described the privacy safeguards (no faces or personal identifiers recorded) and emphasized that participation was voluntary. No customer who objected was photographed; if an image had already been taken, it was deleted immediately. The notices did not disclose the specific hypotheses, stating only that the study examined aggregate consumption patterns.

Cafeteria staff were also briefed about the procedures and trained to provide further clarification upon request. All procedures were conducted in accordance with the ethical standards of the institutional and/or national research committees, as well as the 1964 Helsinki Declaration and its subsequent amendments or comparable ethical principles. Formal approval by an Ethics Committee was not required, as no sensitive or personally identifiable data were collected.

### 2.6. Statistical Analysis

All analyses were prespecified to compare tray selections between the pre-intervention phase (T0) and the post-intervention phase (T1). The analytic dataset comprised 4800 photographed meals (200 trays/day over 12 collection days at each time point). Descriptive statistics were reported as absolute numbers and frequencies, expressed as percentages.

Between-phase differences in categorical variables, overall tray composition, dietary pattern classification (e.g., omnivorous, vegetarian, vegan), composition of first and second courses, side dishes, bread types and accompaniments, fruit/dessert choices, and beverages, were evaluated using Pearson’s chi-square tests with two-sided *p*-values and a nominal α of 0.05. Where cell counts were sparse, Fisher’s exact test was used. Pizza trays were excluded from the analyses that classified dietary patterns and from the assessment of the compliance to the Harvard Healthy Eating Plate [20] and Planetary Diet by EAT Lancet Commission [21], as described in Section 2.4.2.

To appraise the global impact of the nudging intervention on meal quality, trays were classified as compliant or non-compliant with the Harvard Healthy Eating Plate reference [20]; the proportion of compliant trays at T0 vs. T1 was compared using chi-square tests. Negative dietary quality indicators were also operationalized a priori and analyzed as binary outcomes: presence of two animal-protein sources in the same meal, absence of any protein source, absence of vegetables, presence of three carbohydrate sources (pasta/rice *plus* bread *plus* potatoes), absence of fruit, and absence of water (tap or bottled); phase differences in the prevalence of each indicator were tested analogously.

For first-course analyses, subcomponents were coded mutually exclusively as meat, fish, cheese, legumes, vegetables, or plain, and trays were additionally categorized as omnivorous, vegetarian, or vegan (the latter defined as plant-only without added grated cheese). The second course categories included meat, fish, cheese, legumes, and eggs. Side dishes were coded as vegetables, legumes, or potatoes for first and second servings. Bread and accompaniments were classified as white bread, whole bread, breadsticks, or crackers. Fruit/dessert categories included fresh fruit, fruit mousse, yogurt, pudding, fruit juice, and cake; beverages were coded as tap water, bottled water, soft drinks, or beer. All comparisons were conducted on complete cases, and results are presented as *p*-values without multiplicity adjustment, given the primarily confirmatory/monitoring objective of the intervention evaluation. Statistical computations were performed using R (version 2025.05.0+496) statistical software.

## 3. Results

Two hundred trays per day were photographed across 12 days of data collection, both at T0 and at T1, yielding a total of 4800 meals analyzed.

### 3.1. Overall Meal Composition

As shown in Table 1, most trays (>50%) contained only one item for each of the three main components of the meal (first course, second course, side dish). Nevertheless, a proportion of students or customers either omitted at least one component (14.9, 19.7%) or selected more than one item from the same category (0.2, 19.3%). At T1, a significant increase was observed in the selection of a second serving of second courses (*p* < 0.001) and fruit/dessert (*p* < 0.004), whereas the consumption of bread and accompaniments declined (*p* < 0.001).

Overall, the dietary patterns remained relatively stable between T0 and T1, with most trays reflecting an omnivorous profile (Table 2).

### 3.2. Trays’ Components

#### 3.2.1. First Course

Differences emerged in the composition of first courses, with an increased prevalence of dishes prepared with vegetables, cheese, and legumes at T1 (Table 3). The overall distribution of first-course dietary patterns showed a significant shift toward plant-based options (<0.001).

#### 3.2.2. Second Course

By contrast, no significant differences were observed in second-course choices, where meat consistently remained the most frequent option, followed by fish and cheese (Table 4). Also, the overall distribution of first-course dietary patterns showed a high prevalence of animal-based products.

#### 3.2.3. Side Dish

More notable changes were recorded for side dishes (Table 5). Vegetables became the predominant choice in the first serving (53.9%), while potato consumption markedly declined. Legumes also showed a modest increase, although the change was not substantial. Overall, a shift toward greater vegetable consumption in place of potatoes was observed in the post-intervention phase.

#### 3.2.4. Bread and Accompaniments

No substantial modifications were detected in bread and related accompaniments after the intervention (Table 6). White bread remained the dominant choice (75.9%), while whole bread accounted for 24.1%. Crackers and breadsticks were rarely selected at either time point.

#### 3.2.5. Fruit or Dessert

As shown in Table 7, fruit selection increased after the intervention. Fresh fruit was more frequently chosen as both a first option (44.6% at T0 vs. 50.0% at T1) and as an additional option (2.2% vs. 26.9%).

#### 3.2.6. Water and Beverages

Beverage choices are shown in Table 8. Water consistently remained the predominant beverage choice. At T1, an increase in tap water consumption was observed, accompanied by a corresponding decline in bottled water purchases.

### 3.3. Effect of the Nudging Intervention on the Overall Quality of the Meal

After the nudging intervention (T1) the proportion of trays aligning with the Harvard Healthy Eating Plate [20] and EAT-Lancet Planetary Diet [21] models composition significantly increased (<0.001), Table 9.

Based on the dietary quality indicators identified through the Harvard Healthy Eating Plate and EAT-Lancet Planetary Diet frameworks [20,21], the intervention led to clear improvements in several key meal components (Table 10). On one side, a significant reduction was observed in trays containing two different animal protein sources (*p* = 0.037). In addition, trays without a portion of vegetables as a side dish decreased significantly, from 59.2% at T0 to 54.1% at T1 (*p* < 0.001). Likewise, the absence of fruit fell from 65.6% to 10.3% (*p* < 0.001). A significant reduction was also recorded in trays containing multiple carbohydrate sources (37.2% vs. 14.6%, *p* < 0.001). Water consumption remained stable throughout the study period.

## 4. Discussion

The current study demonstrated that nudging strategies can effectively promote nutritionally balanced food choices while simultaneously encouraging environmentally friendly behaviors in a university cafeteria setting. Several types of nudges can be implemented in food environments, and as highlighted in a recent systematic review, presentation, availability, and informational cues represent some of the most promising nudging strategies to foster sustainable food consumption within university cafeterias and similar contexts [47]. In this study, three main approaches were adopted: choice architecture, salient labeling, and educational prompts, supporting the evidence that when healthier items are made more visible and accessible, their selection increases significantly [16,23].

The repositioning of fruit to the central island area led to a substantial increase in its selection, making it the most common dessert choice, while less healthy alternatives such as fruit mousse, or fruit juice declined. Similarly, the label “best choice” highlighting the consumption of vegetables as a side dish proved particularly effective in influencing their selection. Indeed, a notable improvement was observed in the choice of vegetables as side options, accompanied by a marked decline in potato consumption. This trend is particularly relevant in the Italian context, where national surveys indicate that only 44% of students consume one daily serving of fruit and fewer than 25% consume two servings of vegetables per day [48]. Similar findings have been observed in previous nudging interventions, where adjustments to food choice architecture and environmental cues in both school [49] and university [50] cafeterias led to increased selection and consumption of fruit and vegetables, supporting the effectiveness of these strategies across different age groups and settings.

Labels highlighting plant-based dishes had only a modest impact on the selection of first courses containing vegetables or legumes and showed no effect on the variability of second courses, with overall tray composition remaining predominantly omnivorous. The use of labels or symbols to emphasize specific foods is, in fact, controversial in the literature. While some studies have demonstrated their effectiveness [51], others have reported limited or no impact [52,53,54]. In our case, this result may also reflect the fact that the intervention’s primary goal was not to promote vegan diets, but rather to encourage healthier and more balanced meal compositions, particularly through greater inclusion of vegetables, fruits, and appropriate combinations of proteins and carbohydrates. In this sense, overall tray composition improved, suggesting that the educational poster based on the Harvard Healthy Eating Plate composition was effective [20].

However, labels highlighting dishes with legumes did not produce the expected results. Indeed, legumes remained a minority choice among second courses, with even a slight decline after the intervention. Several contextual factors may explain this. First, legumes were offered mainly as side dishes, reinforcing the cultural perception in Italy that they are accompaniments rather than main courses. This reflects a broader inconsistency in dietary guidelines, where legumes are variably classified as protein-rich foods, vegetables, or starchy staples [55,56]. Second, legumes were offered either as plain boiled pulses or as part of soups and stews, preparations that were likely perceived as unappealing or too hot, particularly given the summer season. This limited culinary variety and lack of freshness may have discouraged students from choosing legume-based dishes, despite their nutritional and environmental benefits. Evidence shows that when legumes and vegetables are incorporated into more appetizing recipes, their selection increases markedly [57,58]. These findings point to the need for structural adjustments, such as relocating legumes among main courses and diversifying their culinary presentation, to unlock their potential as sustainable protein alternatives [59].

Bread consumption also illustrates the limits of environmental design. Although whole-meal bread was highlighted as the healthier choice, white bread continued to dominate. This may be due to the lack of clear separation between the two bread types, as previous studies suggest that greater prominence of healthier bread options could enhance their uptake [16,60].

Overall, these results indicate that relatively simple nudging strategies, such as repositioning foods and providing visual prompts, can positively influence students’ choices. Importantly, these interventions are cost-effective, scalable, and adaptable across different institutional settings. Extending them to other university campuses or workplace cafeterias could simultaneously support public health and sustainability goals [61,62]. Future interventions should adopt a multidimensional approach, combining nudging with complementary strategies, such as integrating behavioral cues with nutrition education to enhance awareness, implementing affordability measures to address economic barriers, and extending follow-up periods to evaluate the persistence and transferability of behavioral changes across contexts [61,62]. Involving students in co-design processes, for instance, through focus groups, could further improve the acceptability and effectiveness of these strategies.

The study also presents several limitations. First, analysis was conducted at the tray level, without tracking the same individuals over time, which may have introduced bias in the comparison between baseline and intervention. Moreover, it was assumed that all foods photographed on the tray were consumed, although actual consumption could not be verified. Second, the descriptive design cannot rule out time or menu effects unrelated to the intervention and precludes causal inference, and the short duration of 12 non-consecutive days raises concerns about stability over time. Although menu composition and seasonality could have influenced students’ food choices, both the baseline and intervention phases were conducted during the same season and within a stable menu rotation, minimizing potential confounding effects. Student eating behaviors are strongly influenced by contextual factors such as workload, exams, menu seasonality, and social dynamics, and may also have been affected by participants’ awareness of being observed during tray photography. In addition, we had no information about the students’ general interest in health and environmental issues. As shown in previous studies, visual attention to health-related labels tends to increase healthier choices only among individuals with a high intrinsic interest in health [53]. Third, implementing a control group was not feasible due to practical and logistical challenges, as creating different conditions within the same environment could have disrupted normal operations and introduced unintended variability. To address these constraints and minimize external variability, the study ensured consistent environmental conditions, including conducting data collection in the same location and timeframe. Last, but not least, the study was conducted in a single university cafeteria, limiting generalizability, and no sociodemographic data were collected, which prevented subgroup analyses. These factors suggest caution in interpreting the observed improvements as durable or universally applicable.

Despite these challenges, the FOOD-HACK project also has notable strengths. Conducted in a real-life cafeteria context, it increases ecological validity, reflecting actual consumption rather than hypothetical preferences. The use of multiple nudging strategies, food placement, visibility, and educational tools produced synergistic effects, underlining the value of a multi-component approach. Moreover, by promoting legumes, vegetables, and fruit, the project addressed both nutritional quality and environmental sustainability, aligning with the SDGs. This dual focus is innovative, acknowledging the interconnection between human and planetary health, and moving beyond traditional nutrition-focused interventions [23].

## 5. Conclusions and Prospects

This study shows that small, well-designed nudging interventions can meaningfully improve food choices in university cafeterias. By making healthier and more sustainable options more visible and accessible, institutions can support both public health and environmental objectives. Future work should test the and long-term scalability of these interventions across diverse settings, while integrating behavioral, educational, and structural components. Embedding nudging strategies into institutional food policies, such as collective catering guidelines aimed at providing meals that are not only nutritionally balanced but also environmentally sustainable, state incentives for catering companies to serve plant-based proteins and locally sourced seasonal foods, and cafeteria space design that facilitates healthy choices, could help ensure that behavioral improvements are maintained over time and replicated in other universities or similar contexts.

Moreover, the use of personalized advice, based on a combination of individual characteristics, such as genetic or phenotypic profile and socio-economic and cognitive aspects, would be more effective in guiding consumer choice [63]. Universities, as key institutions shaping young adults’ habits, have the opportunity and responsibility to model sustainable food systems and empower students to make choices that benefit both their well-being and the planet.

## Figures and Tables

**Table 1:** Tray composition at pre- and post-intervention phase.

Course	Pre-Intervention (T0) N = 2400			Post-Intervention (T1) N = 2400			*p*-Value
	0 Dish	1 Dish	2 Dishes	0 Dish	1 Dish	2 Dishes	
**First course** *(pasta, rice or other cereals)*	467 (19.5%)	1907 (79.5%)	26 (1.1%)	473 (19.7%)	1902 (79.3%)	25 (1.0%)	0.9
**Pizza**	2117 (90.7%)	283 (11.8%)	0(0.0%)	2142 (89.2%)	258 (10.8%)	0(0.0%)	0.3
**Second course** *(meat, fish, cheese, legumes or mixed salads)*	362 (15.1%)	2033 (84.7%)	5 (0.2%)	357 (14.9%)	2005 (83.5%)	38 (1.6%)	<0.001
**Side dish** *(vegetables raw or cooked, potatoes)*	372 (15.5%)	1566 (65.3%)	462 (19.3%)	358 (14.9%)	1533 (63.9%)	509 (21.2%)	0.2
**Bread and accompaniments** *(bread, breadsticks or crackers)*	1083 (45.1%)	1304 (54.3%)	13 (0.5%)	1258 (52.4%)	1138 (47.4%)	4 (0.2%)	<0.001
**Fruit or dessert** (*fresh fruit, yogurt, pudding, fruit mousse or pie)*	551 (23.0%)	1803 (75.1%)	46 (1.9%)	496 (20.7%)	1826 (76.1%)	78 (3.3%)	0.004

Data are presented as absolute numbers and frequency, expressed as a percentage. One serving = refers to those who have ordered only one course; two servings = refers to those who have ordered two courses of the same plate (e.g., two first courses or two second courses, etc.).

**Table 2:** Tray composition at pre- and post-intervention phase.

	Pre-Intervention (T0) N = 2117	Post-Intervention (T1) N = 2142	*p*-Value
**Type of meal**			0.3
Omnivorous	1845 (87.2%)	1848 (86.3%)	
Vegetarian	217 (10.3%)	220 (10.3%)	
Vegan	55 (2.6%)	74 (3.5%)	

Data are presented as absolute numbers and frequency, expressed as a percentage. Omnivorous = trays containing meat or fish; Vegetarian = trays containing eggs or cheese (no meat or fish); Vegan = trays containing only plant-based foods (no animal products). Pizza was excluded from this analysis.

**Table 3:** First course.

	Pre-Intervention (T0)	Post-Intervention (T1)	*p*-Value
	Servings N = 1933	Servings N = 1927	
**Added grated cheese**			
Yes	1050 (54.3%)	1004 (52.1%)	0.2
**First course with**			<0.001
Meat	226 (11.7%)	293 (15.2%)	
Fish	637 (33.0%)	481 (25.0%)	
Cheese	361 (18.7%)	373 (19.4%)	
Legumes	42 (2.2%)	48 (2.5%)	
Vegetables	610 (31.6%)	683 (35.4%)	
Plain	57 (2.9%)	49 (2.5%)	
**Type of meal**			<0.001
Omnivorous	863 (44.6%)	774 (40.1%)	
Vegetarian	969 (50.2%)	853 (44.3%)	
Vegan	101 (5.2%)	300 (15.6%)	

Data are presented as absolute numbers and frequency, expressed as a percentage. Omnivorous = trays containing meat or fish; Vegetarian = trays containing eggs or cheese (no meat or fish); Vegan = trays containing only plant-based foods (no animal products, no added grated cheese).

**Table 4:** Second course.

	Pre-Intervention (T0)	Post-Intervention (T1)	*p*-Value
	Servings N = 2038	Servings N = 2043	
**Second course with**			0.12
Meat	1286 (63.1%)	1337 (65.4%)	
Fish	386 (18.9%)	372 (18.2%)	
Cheese	162 (7.9%)	168 (8.2%)	
Legumes	162 (7.9%)	121 (5.9%)	
Eggs	42 (2.1%)	45 (2.2%)	
**Type of meal**			0.026
Omnivorous	1672 (82.2%)	1709 (84.0%)	
Vegetarian	204 (9.9%)	213 (10.3%)	
Vegan	162 (7.9%)	121 (5.7%)	

Data are presented as absolute numbers and frequency, expressed as percentage. Omnivorous = trays containing meat or fish; Vegetarian = trays containing eggs or cheese (no meat or fish); Vegan = trays containing only plant-based foods (no animal products, no added grated cheese).

**Table 5:** Side dish preferences.

	Pre-Intervention (T0)	Post-Intervention (T1)	*p*-Value
	Servings N = 2028	Servings N = 2042	
**First serving**			<0.001
Vegetables	979 (48.3%)	1100 (53.9%)	
Legumes	2 (0.1%)	10 (0.4%)	
Potatoes	1047 (51.6%)	932 (45.7%)	
	**Servings** **N = 462**	**Servings** **N = 509**	
**Second serving**			<0.001
Vegetables	4 (0.9%)	33 (6.5%)	
Legumes	0 (0.0%)	16 (3.1%)	
Potatoes	458 (99.1%)	460 (90.4%)	

Data are presented as absolute numbers and frequency, expressed as a percentage.

**Table 6:** Bread and accompaniments.

	Pre-Intervention (T0)	Post-Intervention (T1)	*p*-Value
	Servings N = 1304	Servings N = 1138	
**Bread**			0.7
White bread	982 (75.3%)	864 (75.9%)	
Whole bread	322 (24.7%)	274 (24.1%)	
	**Servings** **N = 13**	**Servings** **N = 4**	
**Other**			0.5
Breadsticks	7 (53.8%)	3 (90.0%)	
Crackers	6 (46.2%)	1 (10.0%)	

Data are presented as absolute numbers and frequency, expressed as a percentage.

**Table 7:** Fruit or dessert choices.

	Pre-Intervention (T0)	Post-Intervention (T1)	*p*-Value
	Servings N = 1849	Servings N = 1904	
**First serving**			0.001
Fruit	825 (44.6%)	953 (50.0%)	
Fruit mousse	242 (13.1%)	187 (9.8%)	
Yogurt	196 (10.6%)	181 (9.5%)	
Pudding	238 (12.9%)	239 (12.6%)	
Fruit juice	213 (11.5%)	186 (9.8%)	
Cake	135 (7.3%)	158 (8.3%)	
	**Servings** **N = 46**	**Servings** **N = 78**	
**Second serving**			0.001
Fruit	1 (2.2%)	21 (26.9%)	
Fruit mousse	7 (15.2%)	9 (11.5%)	
Yogurt	16 (34.8%)	11 (14.1%)	
Pudding	8 (17.4%)	16 (20.5%)	
Fruit juice	7 (15.2%)	15 (19.2%)	
Cake	7 (15.2%)	6 (7.7%)	

Data are presented as absolute number and frequency, expressed as percentage.

**Table 8:** Water and beverages.

	Pre-Intervention (T0)	Post-Intervention (T1)	*p*-Value
	Servings N = 2400	Servings N = 2400	
**Beverages**			0.87
Tap water	2151 (89.6%)	2198 (91.6%)	
Water Plastic bottle	235 (9.8%)	189 (7.9%)	
Coke or soft drinks	13 (0.5%)	11 (0.5%)	
Beer	1 (0.0%)	2 (0.1%)	

Data are presented as absolute number and frequency, expressed as percentage.

**Table 9:** Trays following the Harvard Healthy Eating Plate and EAT-Lancet Planetary Diet models composition.

	Pre-Intervention (T0)	Post-Intervention (T1)	*p*-Value
	Servings N = 2117	Servings N = 2142	
Adherent	239 (11.3%)	347 (16.2%)	<0.001
Not adherent	1878 (88.7%)	1795 (83.8%)	

Data are presented as absolute numbers and frequency, expressed as percentage. Pizza was excluded from this analysis.

**Table 10:** Negative dietary quality indicators.

	Pre-Intervention (T0)	Post-Intervention (T1)	*p*-Value
	Servings N = 2117	Servings N = 2142	
Presence of two sources of animal protein *trays containing both meat and fish, or two portions of either*	705 (33.3%)	640 (29.9%)	0.037
Absence of protein sources *Trays with no sources of protein such as meat, fish, legumes, eggs or cheese*	36 (1.7%)	40 (1.9%)	0.6
Absence of vegetables *trays with no vegetable side dishes*	1421 (67.1%)	1300 (60.7%)	<0.001
Presence of three carbohydrate sources *trays including a combination of pasta or rice, bread, and potatoes*	893 (42.2%)	351 (16.4%)	<0.001
Absence of fruit *trays with no fruit at the end of the meal*	1575 (74.4%)	1448 (67.7%)	0.001
Absence of water *trays where no water was chosen, either from a refillable bottle or purchased, and only other beverages were selected*	14 (0.7%)	13 (0.6%)	0.8

Data are presented as absolute number and frequency, expressed as percentage. Pizza was excluded from this analysis.

## Data Availability

The raw data supporting the conclusions of this article will be made available by the authors on request.

## Supplementary Materials

The following supporting information can be downloaded at: https://www.mdpi.com/article/10.3390/nu17223562/s1, Table S1: Summer menu adopted during the study period; Figure S1: Plant-based option tag; Figure S2: Dishes with legumes tags; Figure S3: Sustainability poster; Figure S4: Healthy plate poster; Figure S5: Bread flyer; Figure S6: Fruit flyer; Figure S7: Legumes flyer; Figure S8: Herbs and spices flyer; Figure S9: Water consumption flyer; Figure S10: Paper placemat for trays with the Healthy plate.

## Abbreviations

The following abbreviations are used in this manuscript:

Edisu	Ente per il diritto allo Studio Universitario
LDNC	Laboratory of Dietetic and Clinical Nutrition
MD	Mediterranean Diet
NCDs	Non-communicable diseases
OSA	Office for Sustainable Actions
P.I.	Principal Investigator
SDGs	Sustainable Development Goals
