# Supplementary material for: Nudging Healthier and More Sustainable Eating Habits in University Cafeterias: The FOOD-HACK Project
Source: Nutrients. 2025 Nov 14;17(22):3562. doi: 10.3390/nu17223562 (PMC12654996; doi:10.3390/nu17223562)

nutrients MDPI

Supplementary material for "Nudging Healthier and More Sustainable Eating Habits in University Cafeterias: the FOOD-HACK project"

Table S1. Summer menu adopted during the study period.

Day	First courses	Second courses	Side dishes	Fixed alternatives
Monday	• Risotto with scamorza cheese and zucchini†<br>• Cold pasta with raw tomato and mozzarella cheese†<br>• Curry risotto with vegetables†<br>• Mushroom risotto†<br>• Pasta with *arrabbiata* sauce (*hot tomato sauce*)‡<br>• Pasta salad with vegetables‡<br>• Pasta with vegetable ragù‡<br>• Pasta with tomato sauce and clams*<br>• Salmon risotto*	• Cheesy puff pastries†<br>• Potato, ricotta cheese, and spinach pie†<br>• Breaded mozzarella cheese balls†<br>• Chicken escalope with curry*<br>• Mediterranean-style cod fillet*<br>• Rabbit roll*<br>• Chicken escalope with cherry tomatoes and pine nuts*<br>• Beef slice with tomato sauce*<br>• Chicken escalope with artichokes*<br>• Sliced beef*	• Steamed zucchini<br>• Potato croquettes	First courses:<br>• Pasta/rice with oil/tomato sauce‡<br><br>Second courses:<br>• Legumes of the day‡<br>• Cold cuts*<br>• Grilled meat of the day*<br>• Mixed salads with eggs†<br>• Mixed salads with legumes‡<br>• Mixed salads with tuna*<br>• Fresh/aged cheeses†<br><br>Side dishes<br>• Raw vegetables<br><br>Fruit/dessert:<br>• Fresh seasonal fruit<br>• Yogurt<br>• Fruit mousse<br>• Pie<br>• Pudding
Wednesday	• Mushroom risotto†<br>• Spelt and barley salad with pesto, cherry tomatoes, and mozzarella cheese†<br>• Pasta with *norma* (*eggplant and tomato*) sauce‡<br>• Pasta with *Amatriciana* sauce (*tomato sauce and guanciale*)*<br>• Spelt salad with tuna and mozzarella cheese*<br>• Risotto with shrimp, zucchini, and saffron*<br>• Pasta with ragù*<br>• Salmon risotto*<br>• Pasta with tomato, cream, and speck*<br>• Risotto with speck, provola cheese, and chicory*	• Fresh baked ricotta cheese†<br>• Artichoke terrine†<br>• Pizzaiola cheese puffs†<br>• Spicy borlotti bean salad‡<br>• Roast turkey*<br>• Perch fillet with lemon*<br>• Trio of ribs, Prague ham, and pork sausage*<br>• Roast turkey with herbs*<br>• Pork chop with herbs*<br>• Chicken and vegetable salad*<br>• Perch fillet with zucchini and paprika*<br>• Chicken bites with curry*	• Steamed green beans<br>• French fries	
Friday	• Pasta with tomato and pesto sauce†<br>• Risotto with cheese†<br>• Asparagus risotto†<br>• Vegetable risotto†	• Breaded mozzarella cheese balls†<br>• Ricotta cheese and spinach pie†<br>• Vegetable quiche†<br>• Pepper stew with potatoes and peas‡	• Steamed broccoli<br>• French fries	

	• Risotto with peas†	• Meat skewer and chicken sausage*
	• Barley salad with tuna and tomato*	• Mackerel fillet with fresh tomatoes*
	• Pasta with tuna and tomato*	• Pangasius fillet au gratin*
	• Seafood pasta*	• Roast veal*
		• Salmon trout with fresh tomatoes*
		• Roast pork*
		• Perch fillet au gratin*
		• Pork escalope with herbs*

* Omnivorous; † Vegetarian; ‡Vegan

Figure S1. Plant-based option tags.

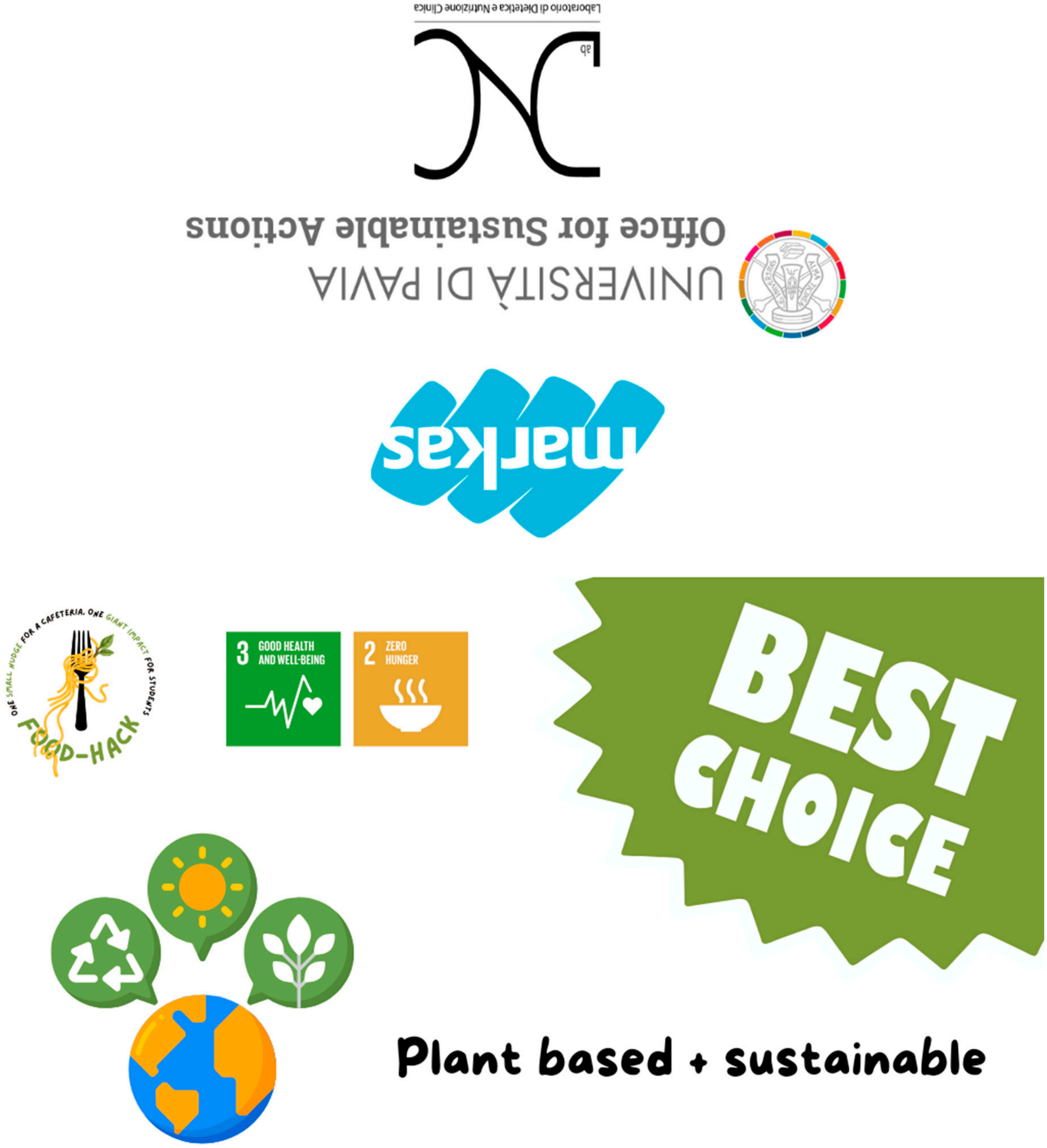

Figure S2. Dishes with legumes tags.

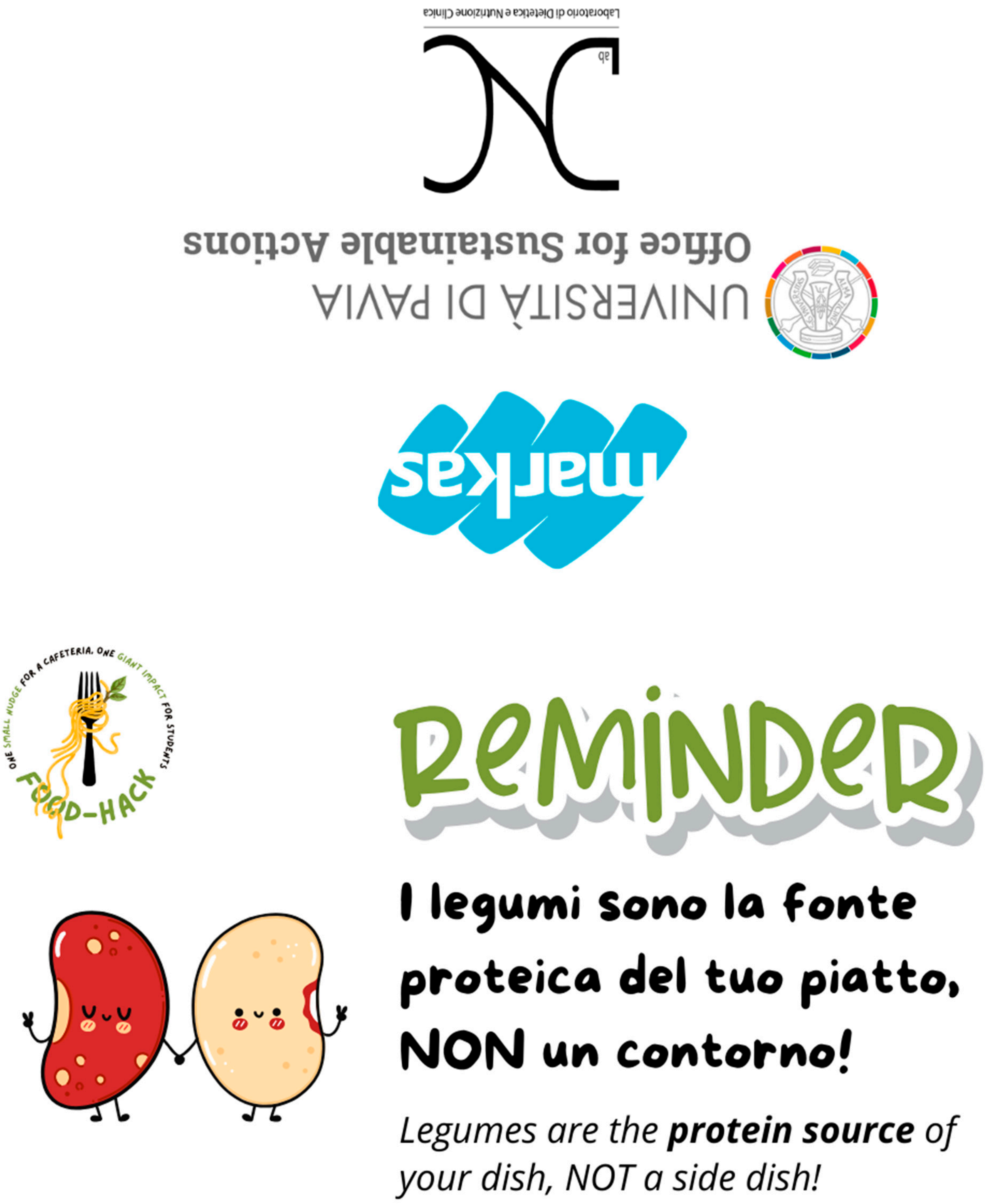

Figure S3. Sustainability poster

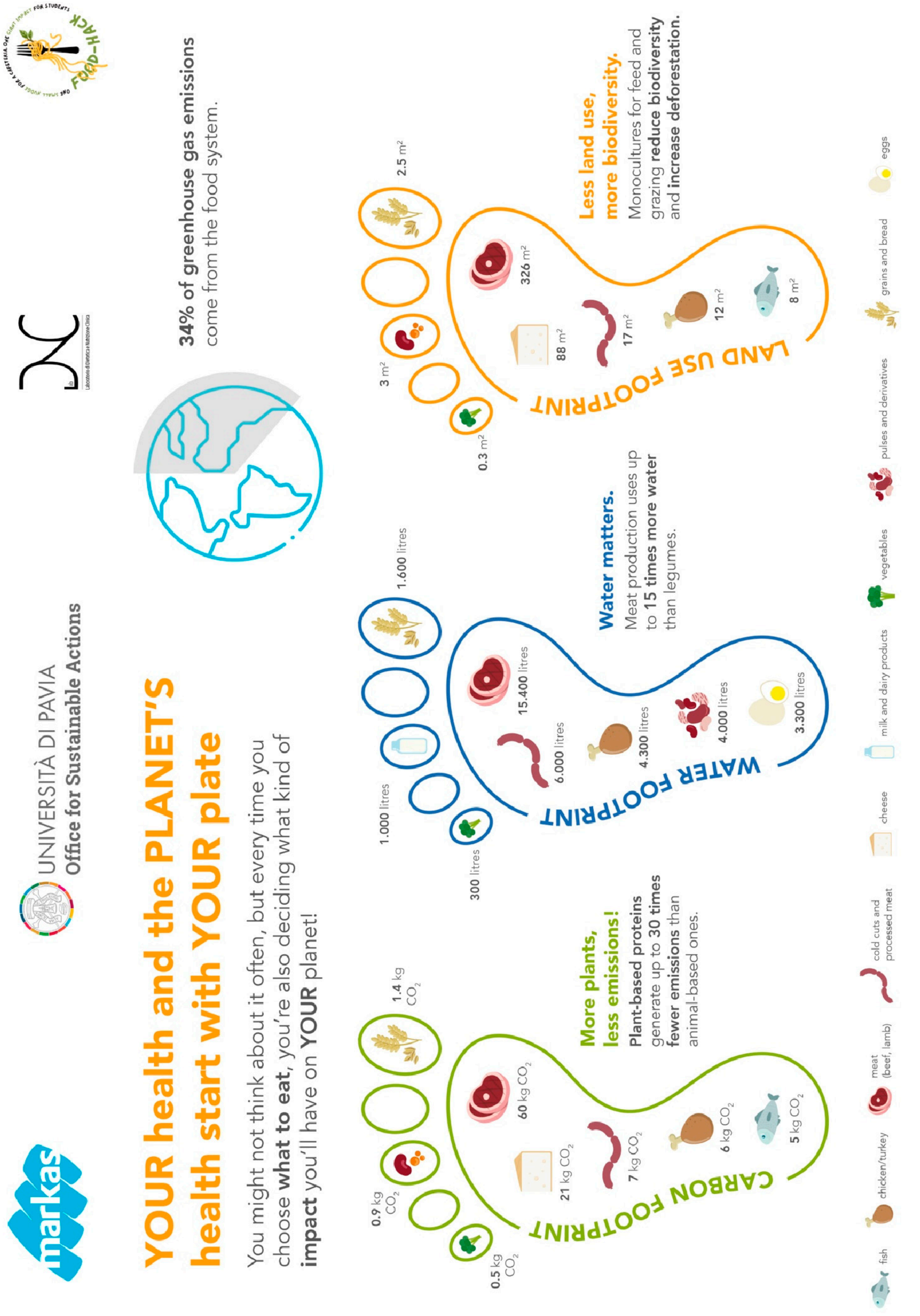

Figure S4. Healthy plate poster

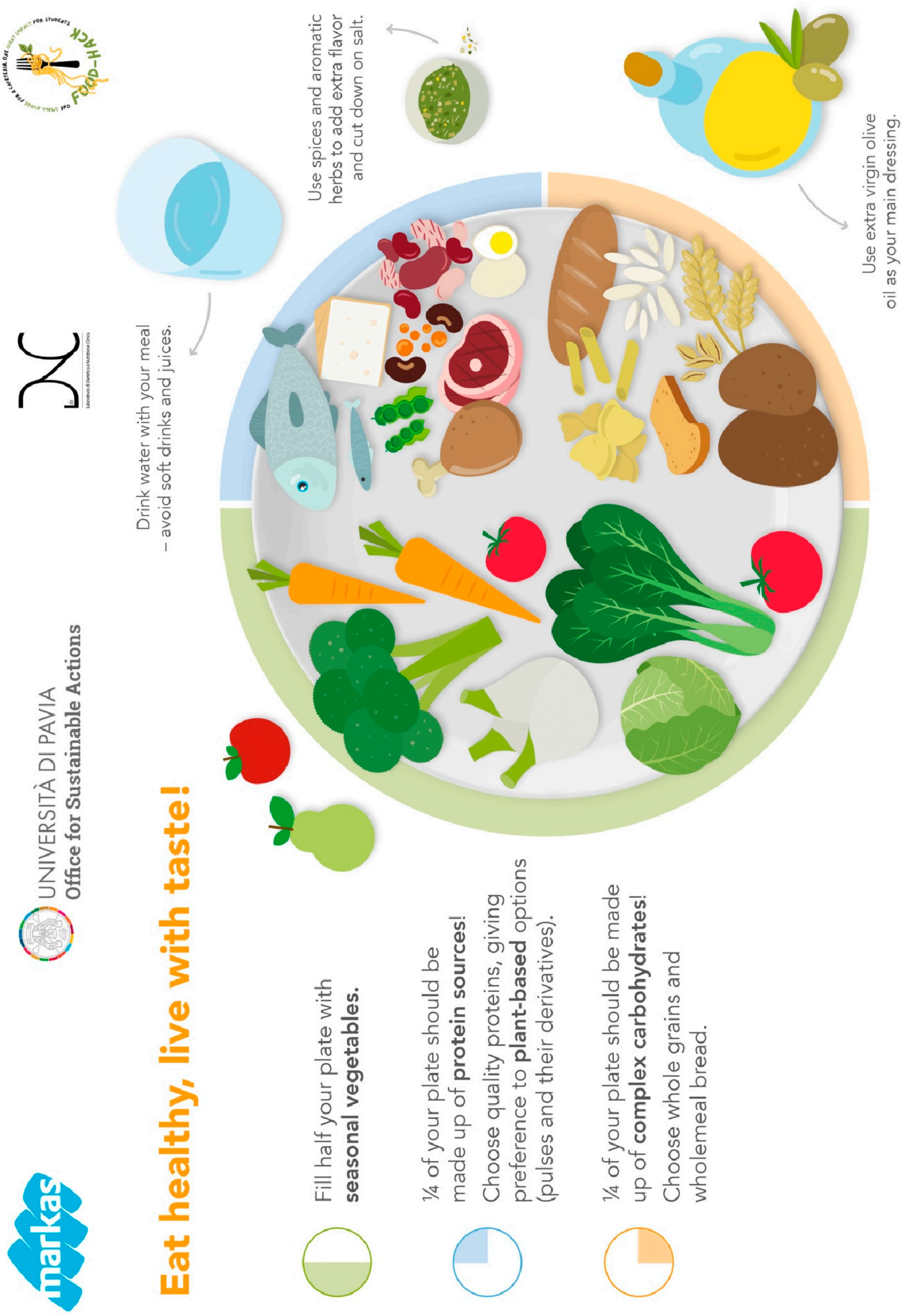

Figure S5. Bread flyer

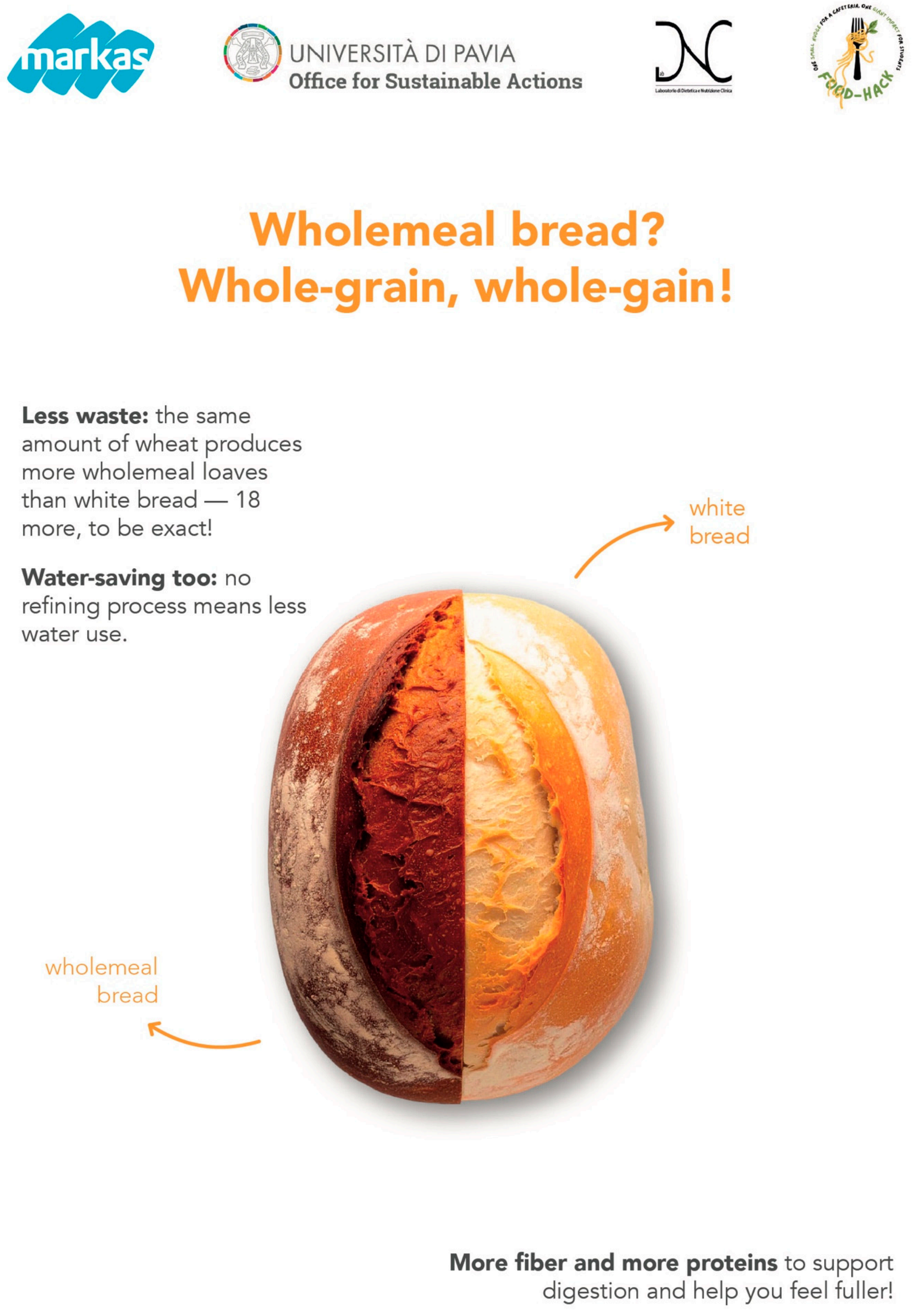

Figure S6. Fruit flyer

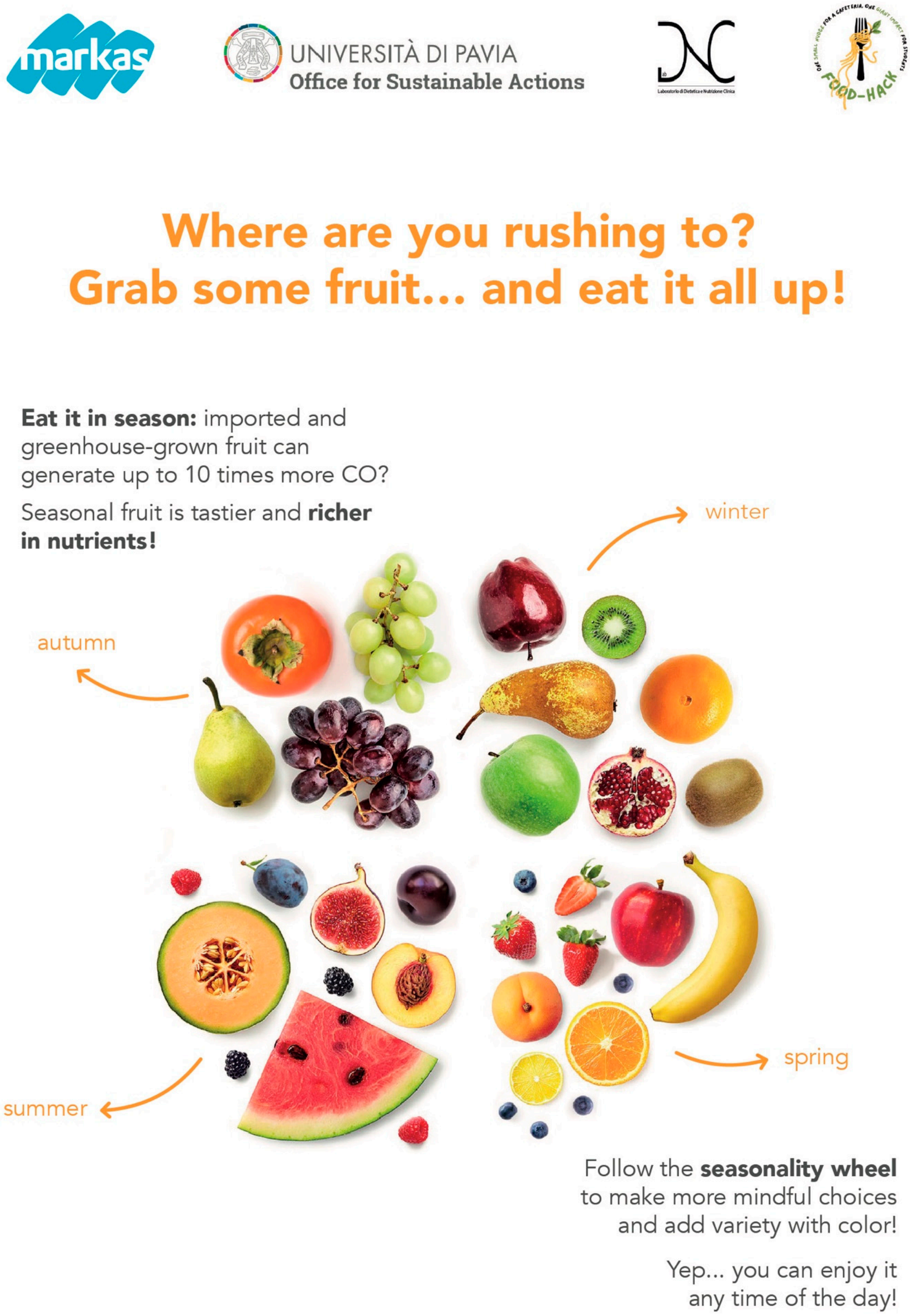

Figure S7. Legumes flyer

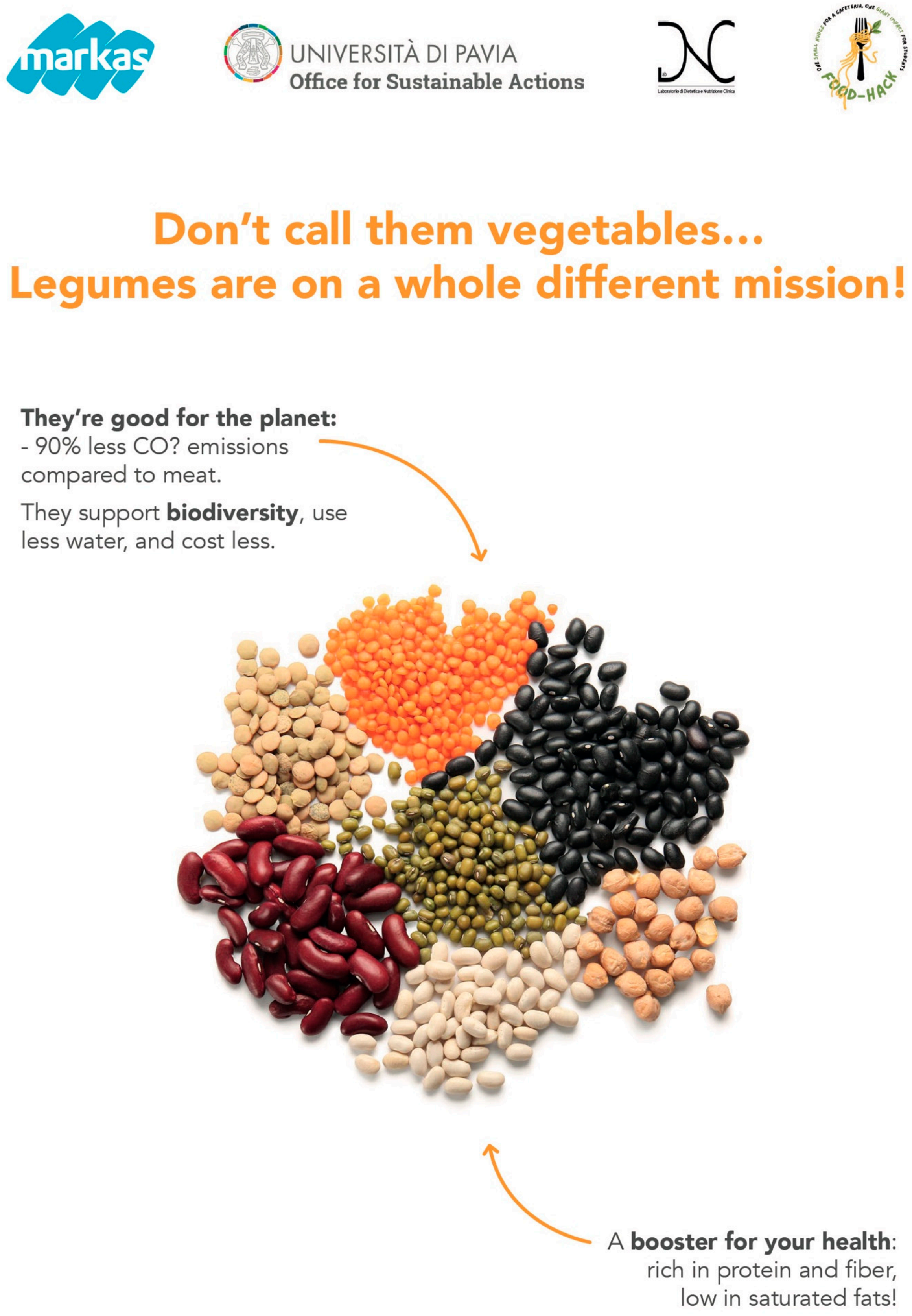

Figure S8. Herbs and spices flyer

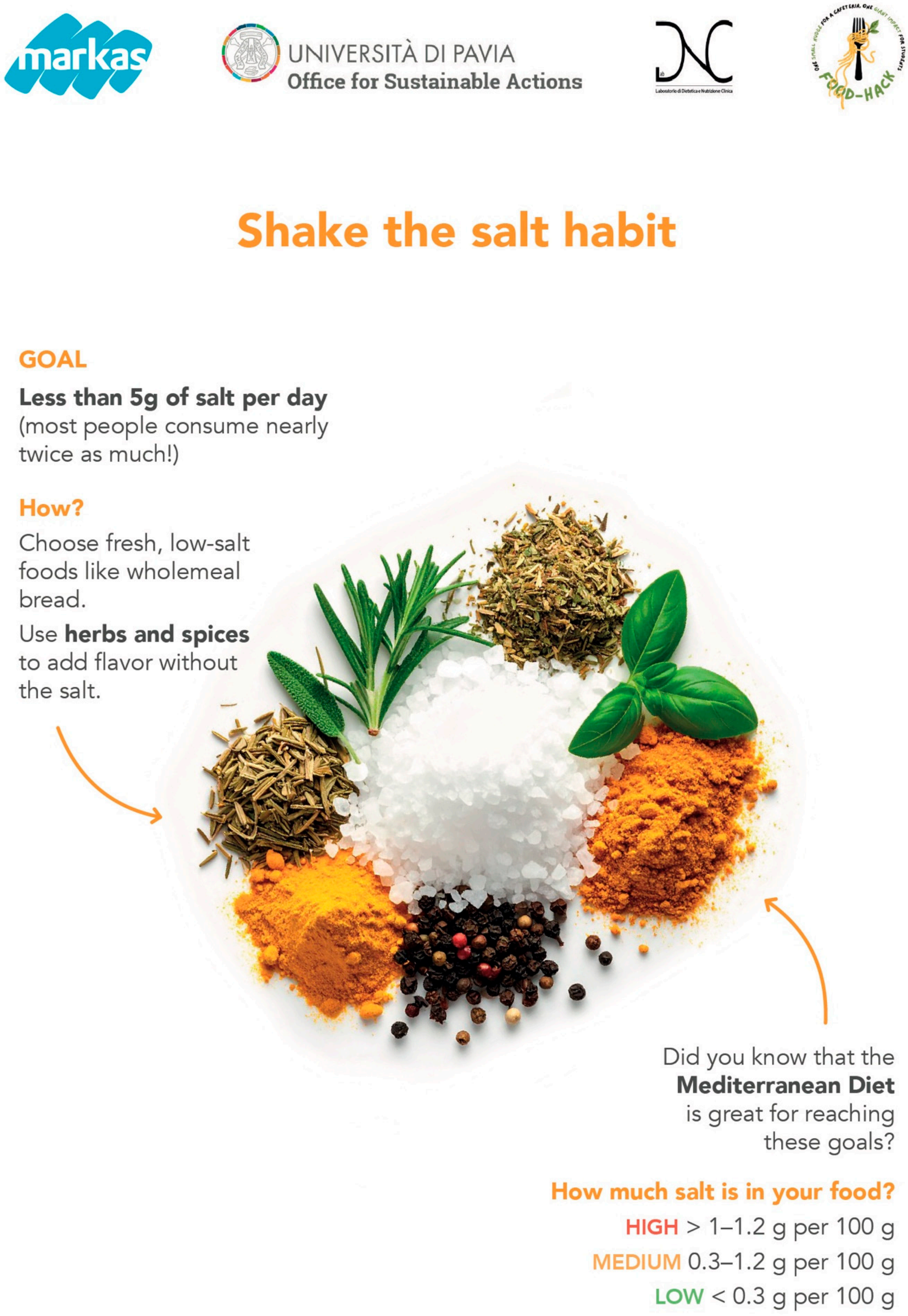

Figure S9. Water consumption flyer.

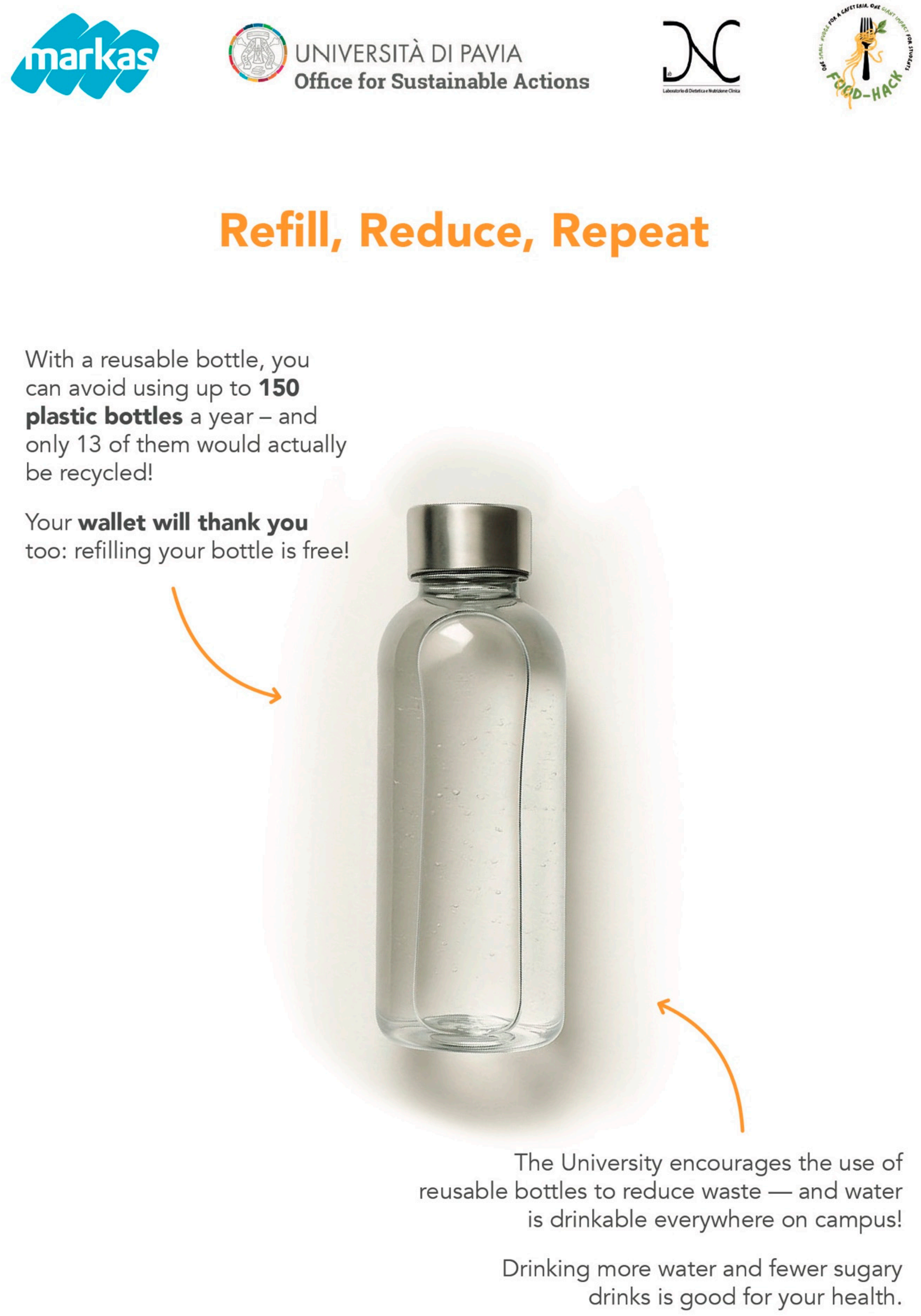

Figure S10: Paper placemat for trays with the Healthy plate.

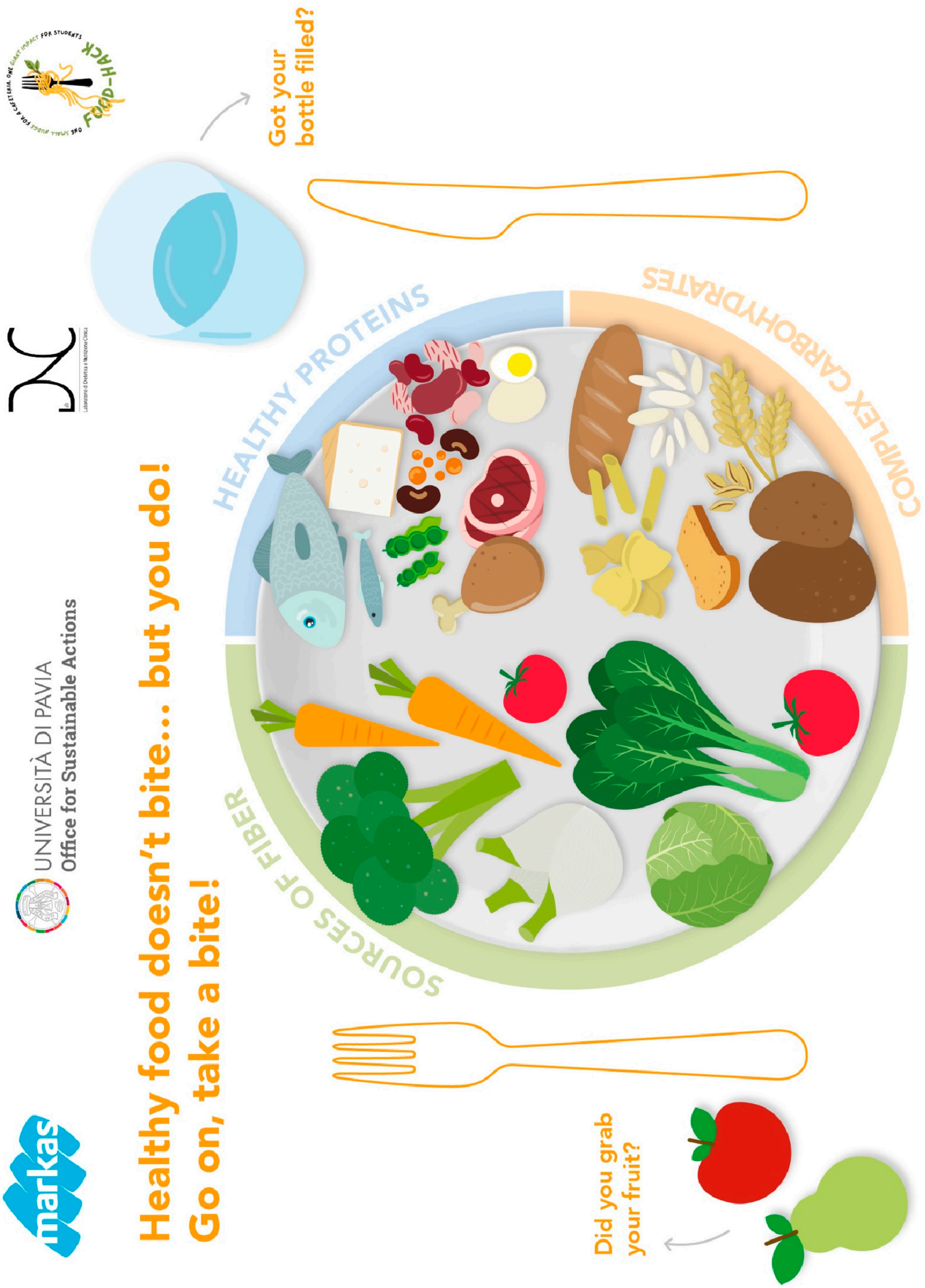

Supplement: Supplementary file 1 [file nutrients-17-03562-s001.zip › nutrients-3971799-supplementary.pdf]
